# Transparent transmission models for informing public health policy: the role of trust and generalizability

**DOI:** 10.1098/rspb.2023.2273

**Published:** 2024-01-24

**Authors:** Soren L. Larsen, Alicia N. M. Kraay

**Affiliations:** ^1^ Program in Ecology, Evolution, and Conservation Biology, University of Illinois at Urbana-Champaign, Urbana, IL, USA; ^2^ Department of Kinesiology and Community Health, University of Illinois at Urbana-Champaign, Urbana, IL, USA; ^3^ Institute for Genomic Biology, University of Illinois at Urbana-Champaign, Urbana, IL, USA

## 1. Introduction

Policy decisions throughout the COVID-19 pandemic have been complex, based on rapidly changing science that, by the time it can be published, may be out of date. This presents a challenge for researchers balancing the advancement of scientific knowledge, communication with the public and government collaboration. The recent work by Conway *et al*. [1] is an exemplar of collaboration between the infectious disease modelling community and government agencies to support policy goals. The authors have developed a thorough model that explicitly incorporates key transitions of interest, including emergency department (ED) visits, hospital admission and ICU stays. The breadth of their parametrization, including not just what was available when the initial government collaboration was conducted but also updated post-hoc analysis with newer Delta parameters, adds confidence to the results. The phases of reopening and key policy goals for these periods are qualitatively clear, providing insight into priorities for policymakers as well as the motivations behind the model. While the results must be considered in light of local context, and may not be generalizable across settings, they provide exceptional insight into the use of vaccine intervention to manage reopening and how models can be used to help guide these decisions. This work also highlights the importance of policymakers setting clearly communicated benchmarks for reopening. In this commentary, we emphasize how carefully selecting and clearly communicating policy targets is crucial for maintaining public trust as well as how findings from a single context might be used to inform decision-making elsewhere. Understanding both the strengths and limitations of this modelling collaboration can inform decision-making as well as future endeavours.

## 2. Transparent models and transparent targets

**Figure 1.**
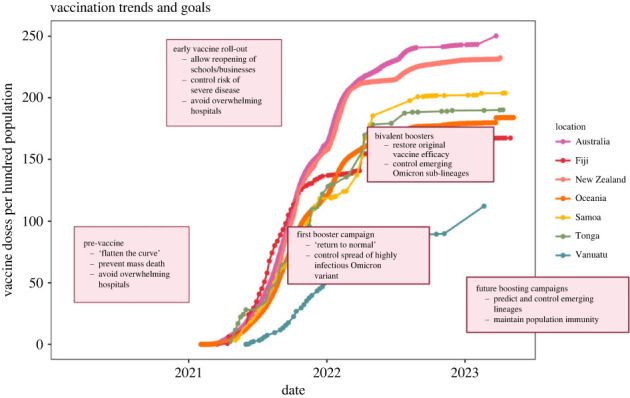
Vaccination trends over time for select Oceanic nations. Key policy goals are highlighted for the pre-vaccine period, early vaccine roll-out, first booster campaigns, bivalent boosters and future booster campaigns. Data sourced from Our World in Data [2]. Figure created with BioRender.com.

Models of disease spread were dominant throughout the COVID-19 response, but the goals evolved over time (figure 1)—from immediate crisis control in the absence of pharmaceutical interventions, towards vaccine policy decisions in a landscape of rapidly changing variants [3]. Regular reports through Imperial College London reveal this transition, beginning with estimation of key disease parameters and hospital capacity in 2020, later shifting to analysis of variants of concern and the impact of vaccine hesitancy in early 2021, and finally the benefit of boosters in late 2021/early 2022 [4]. Reports like these and other sources were instrumental for policymaking throughout the pandemic. Many related results have since been published in the scientific literature (e.g. [5]). By presenting work conducted within the Australian COVID-19 policy framework, the authors join this growing cohort providing ‘under-the-hood’ access to government decision-making.

Sharing models produced for policymakers can help clarify the questions being asked and provide insight into the rationale behind decisions. Illustrating how new information impacts model outcomes may help maintain public trust—an issue of key importance throughout the pandemic, where distrust rose swiftly on the heels of wavering on mask mandates and routes of transmission. This is especially true when the results of those decisions are not as successful as hoped and leave lingering questions. For example, how could a country with universal healthcare and an early response experience such a high burden of mortality compared to its peers? For Sweden, the answer is likely highly complex and not due to policy alone [6]—highlighting that a policy post-mortem can point to the influence of other important factors on disease dynamics. Modellers often contend with data that lags behind reality, as Conway *et al*. show through their *post hoc* analysis for the Delta wave. Encouragingly, their results hold even when updated to account for the increased severity of the Delta variant. Illustrating how new information impacts model outcomes may help maintain public trust.

One of the most important considerations to arise from this work is that modellers need clear guidance from policymakers on the goals of intervention as well as thresholds for optimal disease control, particularly in the move from transmission blocking to suppression.

With regard to thresholds, the authors have provided strong qualitative grounding for the vaccination thresholds identified in the analysis, in concert with the phases and control measures of Australia's National Plan. They also present useful examples of how mitigation strategies can mix-and-match, and be stacked to achieve complete (or enhanced) disease control. The flexibility of stacking or revising interventions throughout the simulation period is a core strength of this work. However, while the authors discuss the ideal level of coverage for reopening, agreed upon in consultation with policymakers, the underlying transmission and mortality thresholds are never explicitly stated or justified. With limited quantitative benchmarks, it is hard to determine from the figures alone, for example, why 70% vaccine coverage is preferred to 60%. The authors also do not address why it is preferable to vaccinate at 60% coverage and prioritize older adults, instead of vaccinating 50% of the population through an ‘all adults’ strategy, when the transmission curves are so similar. Asking policymakers, who are often not subject-matter experts, to define thresholds *a priori* comes with its own set of challenges—goals may be unrealistic, communicated through elusive metrics or difficult to project within the limitations of a given model. In cases such as this where the model-generated data do not clearly illustrate a preferred strategy, adding additional clarity in the text about how the ultimate policy decision was selected, even if quantitative factors were not the final source, would be helpful.

This disconnect may reflect, in part, the importance of other policy objectives that are not as easily quantified by the standard transmission model outputs of cases and deaths, such as reducing steep economic and social costs of lockdowns that may themselves lead to disease and mortality, albeit less directly. In many high-income countries, including Australia, where implementing lockdowns was more financially feasible, minimizing deaths due to COVID-19 was the primary policy objective until vaccines were available, irrespective of the resulting economic costs of this policy (figure 1). However, in other parts of the world, the economic and social costs of the pandemic response were also a concern that shaped policy and compliance with those policy recommendations sooner. In many low- and middle-income countries, for example, initial lockdowns could not be sustained due to their social and economic costs, which could not readily be mitigated given the lack of available infrastructure to support continued access to work opportunities (e.g. through telework) and education (e.g. through distance learning) during lockdown [7]. In low-income countries in particular, strict adherence to social distancing also had health implications, such as increased risk of starvation due to lost income [7].

While these costs were not generally as extreme in high income countries, social considerations were also a factor in some high-income countries even at the start of the pandemic. For example, the Swedish mitigation strategy reflected, in part, a priority on equity—though it was unsuccessful in eliminating inequities in the distribution of mortality [8]. While detailed economic models are beyond the scope of traditional transmission modelling work, social costs can still be quantified to an extent through other means. When policy goals are clearly communicated to modellers, this information can also be clearly presented at the time of publication so that the public can understand why the government chose a particular strategy that may not seem optimal from a health outcomes perspective.

Defining clear, quantitative metrics is a challenge not just in this work but in modelling at large. ‘Disease control’ and what it constitutes will vary by place and time; across healthcare access, economic capability, and political will. Optimal control strategies can depend on whether transmission, healthcare capacity or mortality is being targeted (e.g. [9,10]). Where some models are more equipped to answer certain questions than others, having a clearly defined target is crucial to being able to adequately capture relevant disease dynamics and convey results to policymakers. Still, Conway *et al*. have produced a strong example of the utility of modellers and policymakers working together to tackle complex problems in infectious disease.

## 3. Applicability versus generalizability

There is a balance to strike between local versus global models, and age-based vaccine prioritization has already been strongly explored in generalized communities [10]. Providing projections solely for synthetic communities may limit translatability into policy and decrease relatability for the public. The local demographic structure of a community may be highly influential on optimal vaccine prioritization, as has been previously demonstrated within the context of the USA [9]. For these reasons, it is exciting that Conway *et al*. have situated their work within a local context. The fact that their modelled scenarios were generally consistent with observed transmission patterns under the rollout scenarios that were implemented, adds confidence that the results are robust.

By grounding their model in Australia, Conway *et al*. increase the applicability of the findings within a local setting and strengthen its utility for decision-makers. In particular, by explicitly addressing ED visits, hospital admissions and ICU admissions in their model, they create a suite of indicators for Australian policymakers to choose from during decision-making about vaccine coverage thresholds. A question that could be explored further in the future is the risk of exceeding domestic hospital capacity. While the authors consider ICU utilization in this work, they do not compare to national or regional hospital resources. However, they have also modelled jurisdictional-level healthcare capacity for the Australian government in the past [11]. Nevertheless, given that the severity and transmissibility of circulating variants has changed throughout the pandemic, relating current and projected future incidence to these thresholds would be useful. To revisit the point of quantitative thresholds, characterizing levels of mitigation that would result in overwhelming healthcare infrastructure is a useful tool for decision-makers—though it can be deceptively challenging to quantify due to changes in healthcare capacity over time and across jurisdictions. Further consideration of within-country heterogeneity may be important for future work. Still, Conway *et al*. [1] provide comprehensive, location-focused metrics.

Although specific contexts can be very useful, the optimal control strategy might vary by site due to differences in transmission dynamics and the feasibility of specific control targets. Geographic isolation is associated with reduced pathogen species diversity, and in the context of measles, influences endemicity [12]. It is possible that for SARS-CoV-2, the dynamics of an island nation like Australia may impact transmission and optimal control. For example, Iceland, another high-income island nation, responded somewhat reactively to the COVID-19 outbreak and still managed to control the outbreak effectively through a testing-based strategy [13]. The authors acknowledge that Australia was already a low-transmission area before vaccine-rollout. In considering vaccine coverage targets, relative wealth is also a factor; globally, low-income countries experienced lower rates of vaccination than high income countries during primary series rollout, such that 60–70% coverage may have been unattainable for much of the Global South [14]. Comparing Australia's vaccination trends over time to that of some of its near neighbours in Oceania, it is clear that Australia consistently has the highest vaccine coverage (figure 1). With its stark geographical boundaries and a high GDP, it is somewhat of an outlier.

In thinking about applicability, policymakers must be cautious not to extrapolate findings from one locale to another without consideration of heterogeneity. Across fundamentally different places, with varied demographics, behaviour, social and environmental conditions, the same model might predict fundamentally different dynamics if the key aspects of heterogeneity are not accounted for. The strength in country-specific models—focusing on one context to provide highly detailed results—can also be a weakness. Great care should be taken when applying country-specific findings to policy decisions in another context, both at international and subnational scales. Even within a country, the distribution of disease burden could be highly heterogeneous, for example, by race [15] or socioeconomic status [16], but this might be obscured by the model construction.

## 4. Future directions

Conway *et al*. [1] have provided a guiding example of the ways in which policymakers and modellers can work together to inform robust decision-making. The presentation of clear policy goals and intervention strategies, with simulations conducted in the Australian context, provide a roadmap for future collaborations. Future work might include more quantitative guidelines from policymakers. Additionally, when models are being designed to project metrics for specific countries, consideration of within-country heterogeneity—from jurisdictional capacity to sociodemographic characteristics of the population—can help to provide a better understanding of the distribution of disease burden.

## Data Availability

All data used to make figure 1 are publicly available from Our World in Data (https://ourworldindata.org/coronavirus).
